# Equity aspects of the Primary Health Care Choice Reform in Sweden – a scoping review

**DOI:** 10.1186/s12939-017-0524-z

**Published:** 2017-01-28

**Authors:** Bo Burström, Kristina Burström, Gunnar Nilsson, Göran Tomson, Margaret Whitehead, Ulrika Winblad

**Affiliations:** 1 Department of Public Health Sciences, Equity and Health Policy Research Group, Karolinska Institutet, SE 171 77 Stockholm, Sweden; 2 Department of Learning, Informatics, Management and Ethics, Health Outcomes and Economic Evaluation Research Group, Karolinska Institutet, Stockholm, Sweden; 3 Department of Neurobiology, Care Sciences and Society, Karolinska Institutet, Stockholm, Sweden; 4 Department of Learning, Informatics, Management and Ethics, Karolinska Institutet, Stockholm, Sweden; 5 Department of Public Health and Society, Institute of Psychology, Health and Society University of Liverpool, Liverpool, UK; 6 Department of Public Health and Caring Sciences, Health Services Research, Uppsala University, Uppsala, Sweden

**Keywords:** Equity, Inequalities, Health care need, Primary Health Care Choice Reform, Quality of care, Reimbursement system, Resource allocation

## Abstract

**Background:**

Good health and equal health care are the cornerstones of the Swedish Health and Medical Service Act. Recent studies show that the average level of health, measured as longevity, improves in Sweden, however, social inequalities in health remain a major issue. An important issue is how health care services can contribute to reducing inequalities in health, and the impact of a recent Primary Health Care (PHC) Choice Reform in this respect. This paper presents the findings of a review of the existing evidence on impacts of these reforms.

**Methods:**

We reviewed the published accounts (reports and scientific articles) which reported on the impact of the Swedish PHC Choice Reform of 2010 and changes in reimbursement systems, using Donabedian’s framework for assessing quality of care in terms of structure, process and outcomes.

**Results:**

Since 2010, over 270 new private PHC practices operating for profit have been established throughout the country. One study found that the new establishments had primarily located in the largest cities and urban areas, in socioeconomically more advantaged populations. Another study, adjusting for socioeconomic composition found minor differences. The number of visits to PHC doctors has increased, more so among those with lesser needs of health care. The reform has had a negative impact on the provision of services for persons with complex needs. Opinions of doctors and staff in PHC are mixed, many state that persons with lesser needs are prioritized. Patient satisfaction is largely unchanged. The impact of PHC on population health may be reduced.

**Conclusions:**

The PHC Choice Reform increased the average number of visits, but particularly among those in more affluent groups and with lower health care needs, and has made integrated care for those with complex needs more difficult. Resource allocation to PHC has become more dependent on provider location, patient choice and demand, and less on need of care. On the available evidence, the PHC Choice Reform may have damaged equity of primary health care provision, contrary to the tenets of the Swedish Health and Medical Service Act. This situation needs to be carefully monitored.

## Background

In many countries, including Sweden, primary health care (PHC) is the basis of the health care system and contributes in an important way to the improvement of health in the population [1]. Good health and equal health care are the cornerstones of the Swedish Health and Medical Service Act [2]. Recent studies show that the average level of health, measured as longevity, improves in Sweden, among men and women [3]. However, social inequalities in health remain a major issue, new health divides are surfacing and the Government has recently commissioned an investigation into how inequalities in health can be reduced [4]. An important issue is how health care services can contribute to reducing inequalities in health, and the impact of recent reforms in PHC in this respect. This paper presents the findings of a review of the existing evidence on impacts of these reforms, as well as identifying the gaps in the current literature that need to be addressed by new empirical studies.

### The Swedish welfare state and health care system

Swedish welfare services, including health care services, were developed after World War II, with the aim of creating a comprehensive public system for provision of services, of high quality and universally accessible for all [5, 6]. The notion of providing good quality care services to the whole population has been regarded as part of the public welfare system in Sweden, providing high quality health care, schools, elder care and other social services to the entire population [5]. A universal welfare system, such as the Swedish health care system, requires the loyalty of the middle class in order to gain legitimacy and be sustained [7]. This in turn means that the services have to be of high quality to satisfy all users, an idea referred to by Rothstein [8] as “the high-quality standardized solution”. If successful, the system could then as intended promote egalitarianism and social integration.

The Swedish health care system is tax funded and the responsibility for it is decentralized to county councils and regions, which collect taxes for the purpose [5]. As other parts of the welfare system, the health care system is based to a great extent on egalitarian principles, and the national level provides legislation and guidelines for health care. The main objective for Swedish health care, as expressed in the Health and Medical Services Act [2] from 1982, is good health in the entire population and health on equal terms, and equitable care based on need. The bulk of health services have been operated by public providers, but in recent years the proportion of private (for-profit) providers has increased, particularly in outpatient care and PHC [5].

The core function of Swedish PHC is to be the first health care contact for the population, to provide holistic and comprehensive care, ranging from health promotion, disease prevention and curative care to rehabilitation. PHC deals with the whole population, the whole individual, the entire disease panorama, and the disease process over the entire life course of individuals. This distinguishes PHC from secondary and tertiary care, which provide important services at specific occasions of patients’ diseases.

Public PHC centres in Sweden have been established in a planned manner in local residential catchment areas to serve the population, and typically include 4–10 general practitioners (GPs), nurses, other paramedical professionals (physiotherapists, occupational therapists, podiatrists). In addition many PHC centres provide maternal and child health services. PHC also includes outreach services, not least through district nurses, to patients who need such services, and interacts with other public authorities [5]. PHC also offers on-site medical care services to residents in local nursing homes. Although not a gate keeper to secondary care as in some other countries, PHC is the first point of contact for most patients in Sweden who end up in secondary care [5].

The proportion of GPs and level of resources going to PHC in Sweden is lower compared to other high-income countries [9]. However, in a recent Swedish government investigation, a well-functioning PHC was mentioned as “probably the single most important activity by which the health care system can contribute to improve equity in health” ([10] p 375).

In international comparison, the average number of outpatient visits (to PHC as well as to specialist outpatient services) is considerably lower in Sweden than in other countries. In Sweden (and Finland) the average number of visits is about three per person and year, compared to about six for OECD countries [9]. This may be due to the organization of health care in Sweden, where a greater number of visits are done by nurses, and to the fact that Swedish health care is dominated by hospital based care. Nevertheless, access to outpatient care is considered to be lower in Sweden than in other countries.

### Choice reform and market orientation in primary health care

In recent years market orientation has increased in the Swedish health care system as a whole. A national law on freedom of choice by citizens was passed in 2008 [11], to enable citizens to choose among providers in different sectors, including health and social care [12]. In 2010 an amendment was made to the Health and Medical Services Act, mandating the regions and county councils to allow citizens to choose their PHC provider, and to allow private providers of PHC to freely establish practices, if they met certain defined criteria. The objectives of this PHC Choice Reform were to increase patient choice, expand the provision of private health care to increase access to care, and to increase quality and innovation through competition among providers [11–13].

The law on freedom of choice was enforced by a centre-right government and there have been differing ideological views on the benefits of the PHC Choice Reform. The PHC Choice Reform implies a shift from an egalitarian towards a libertarian ideology in health care [14]. An analysis of policymakers’ arguments when the PHC Choice Reform was legislated focused on whether and how the PHC Choice Reform harmonizes with the emphasis on equity in the Health and Medical Services Act, which population groups will actually benefit from the reform, and ultimately how the reform may impact on the role of PHC on population health and inequalities in health [15]. The study concluded that because health inequalities is one of the main challenges, the impact of health care reforms on equity should receive more attention in policy making [15].

Theoretically, the reform may impact in different ways on equity aspects of PHC. On the one hand, access to care may be increased for all by allowing free establishment of providers; on the other hand, the free establishment may result in providers choosing where to establish, and reduce political opportunities for deliberate need-based resource allocation between PHC centres. In addition, the Choice Reform may impact on the role and assignment of PHC and thereby the prioritization of patients and the work of GPs. The organization of work and prioritization of patients in PHC is further affected by the type of financial reimbursement system employed, and how different PHC activities are incentivized. The reform has also subsequently been implemented differently in different county councils [14]. However, there is little scientific evidence on the impact of the reform.

Donabedian’s framework for assessing quality of care [16], which distinguishes three aspects of quality in care: structure, process and outcomes, may be used as a point of departure for the analysis. In Donabedian’s framework, structure refers to the settings in which care occurs, including facilities, equipment and monetary resources, human resources and organizational structure such as staff organization and methods of reimbursement. Process describes what is done in health care, including the patient’s seeking care as well as the practitioner’s activities in diagnosing and treating the patient. Outcome denotes the effects of care on health status of patients and populations, also including the patient’s satisfaction of care [16].

In view of the emphasis on equity in Swedish health care policy, one important research question is how the PHC Choice Reform and increased market orientation will affect PHC in terms of equity aspects on structure, process and outcome of PHC. In particular, the reform may influence: 1) The establishment of new PHC clinics and resource allocation between PHC clinics, 2) The organization and implementation of PHC and how different patient groups are prioritized, and 3) The impact of PHC on population health.

The aim of this study was to review and analyze the evidence regarding the equity impact of the PHC Choice Reform in Sweden.

## Methods

We did a scoping review of the published accounts (scientific articles and reports) which reported on the impact of the Swedish PHC Choice Reform of 2010 and changes in reimbursement systems, from 2008 to September 2016. From a search on PubMed and Web of Science we found six scientific articles. We also searched for “grey literature” including publications from relevant public agencies in Sweden concerning the PHC Choice Reform and found nine publications, three with a nationwide focus (one with in-depth analysis of data from three county councils), two covering three county councils and four reports were based solely on data from Stockholm County Council where the reform was introduced already in 2008, before it was legislated nationally in 2010.

The results of the review are organized according to Donabedian’s framework of structure, process and outcome. The results are summarized in a narrative manner.

## Results

The review resulted in 6 scientific articles and 9 reports, which are presented in Table 1. The main objectives of the PHC Choice Reform were to increase patients’ choice of PHC provider, expand the provision of privately provided health care and increase quality and innovation through competition among providers [12]. An overview of the effects of the PHC Choice Reform based on Donabedian’s framework and different reimbursement systems is presented in Table 2.

**Table 1:** List of reviewed publications

Publication	Ref. no.	Year	Area(s)	Data	Focus	Results
Scientific articles						
Beckman, Anell	[23]	2013	Region Skåne	Population register data	Process of care – PHC visits	Visits increased more among high-income than low income earners
Agerholm et al	[25]	2015	Stockholm County council	Population register data, public health survey data	Process of care – PHC visits	Visits increased more among person with lesser needs; less among those with greater needs
Glenngård	[32]	2013	Region Halland, Skåne, Västra Götaland	Patient survey data	Outcome – Patient satisfaction	Satisfaction with primary care higher in areas with low level of social deprivation and in smaller practices
Maun et al	[27]	2013	Gothenburg	Interviews with 24 PHC managers	Process of care – doctors’ views	Prioritisation conflicts among doctors between patients with different needs and demands. Chronically ill patients were crowded out.
Hollman et al	[28]	2014	Gothenburg	Interviews with PHC district nurses	Process of care – nurses’ views	Reimbursement system emphasizes doctors and plays down nurses’ role. Negative for job satisfaction and work environment
Isaksson et al	[17]	2016	Nationwide	Area socioeconomic composition of population in relation to established clinics	Structure – establishment of new practices	New centres located in areas with fewer old adults living alone and fewer single parents. No significant effects of income, percentage immigrants, education, unemployment
“Grey literature”						
Rehnberg et al	[21]	2008	Stockholm County council	Population register data	Visits, productivity, resource allocation	Increase in visits and in productivity overall. Resources decreased in areas with greater need
Glenngård	[31]	2012	Region Halland, Region Skåne, Region Västra Götaland	Patient survey data	Outcome - Patient satisfaction	Satisfaction with primary care higher in areas with low level of social deprivation and in smaller practices
Swedish Association of Local Authorities and Regions	[19]	2012	Nationwide	Survey among 360 PHC managers	Doctors’ views on reform and reimbursement systems	Dissatisfaction with reimbursement systems, leading to prioritization of patients with lesser needs
Johansson	[30]	2012	Stockholm County Council	Survey among PHC doctors and nurses	Health promotion in PHC	Negative impact on health promotion because of lack of reimbursement
Dahlgren et al	[24]	2013	Stockholm County Council	Population register data	Visits, patient satisfaction, new practices	Increase in visits for all but more among high income earners. Patient satisfaction generally not affected, but lower among patients with greater needs. New practices spread out.
Mohmand	[29]	2014	Stockholm County Council	Interviews with 6 PHC doctors	Process of care – doctors’ views	PHC reform makes patients to be customers Reimbursement system prioritises those with lesser needs
National Audit Office	[18]	2014	Nationwide, three county councils in-depth	Population register data, interviews	Structure – establishment of new practices	More new practices in wealthy larger urban areas, interviews suggesting practitioners not establishing in areas with greater need
Myndigheten för vårdanalys	[26]	2015	Stockholm County Council, Region Västra Götaland, Region Östergötland	Population register data	Process of care – PHC visits	Increase in visits among all, stronger among high income earners. Higher increase among person with no diagnosis indicating higher health care need.
Government investigation “Efficient care”	[10]	2016	Nationwide	Meetings, interviews, documents	Organization of health care, role of PHC	PHC very important to the whole health care system, should be first line for all. PHC Choice Reform has made cooperation around patients with complex needs more difficult. Suggest legislation for separate organization of PHC for these patients.

**Table 2:** Overview of potential and observed effects of the PHC Choice reform and reimbursement systems on structure, process and outcome in PHC in Sweden

	PHC Choice Reform	Reimbursement system based on fee-for service	Comments - impact on equity and need-based care
Structure - Access, resources			
Number of practices	Increased		Less increase in disadvantaged areas
Practice distribution	Providers’ choice determines practice distribution		Reduced political influence on distribution by need, may cause maldistribution
Resource allocation	Patients’ choice determines resource allocation between practices	Short visits are incentivised = more income	Reduced political influence on resource allocation by need
GP’s work environment	Patients become customers - change in professional focus	Many short visits are incentivised	Priority on those with lesser needs
Process - Delivery of health care			
Number of visits to PHC	Increased	Increased	Greater increase for those with lesser needs
Prioritisation of patients	Patients as customers	Focus on short visits by healthier patients	More demand-driven care. Less focus on those with greater need
Integrated care	More complex to achieve integration, competition	Integrated care not incentivised	More difficult for those in need of integrated care
Holistic care	De-limited, differentiated PHC assignments (e.g. ENT, gynaecology, child health)	One visit, one problem (short itemized visits)	Itemized care not beneficial for those with complex needs
Inter-professional care	Focus on doctors	Less teamwork doctors and nurses	No benefit for those in need of inter-professional care
Outcomes – impact on health			
Health among those with complex needs	Coordination and integration more difficult	Counteracts holistic care	Potentially adverse effects on those with greater needs
Treatment impact	Reduced focus on prevention, more emphasis on cure	Focus on short visits - curative care for self-limiting diseases	Increase in preventable health problems?
Population health	Focus only on listed individuals limits population impact	Less emphasis on health promotion and on collaboration with other agencies	Reduces PHC impact on population health

### Effects on the structure of PHC – new facilities and reimbursement

#### Effects on the establishment of PHC facilities

Since 2010, over 270 new private PHC practices have been established throughout the country, operating for profit [17]. In 2014 the Swedish National Audit Office presented an investigation of the PHC Choice Reform [18]. Their report concluded that the number of PHC clinics had increased in 20 out of 21 county councils, but that the new establishments had primarily located in the largest cities and urban areas, in socioeconomically more advantaged populations [18].

In contrast, another recent study [17] which adjusted for effects of county council regulation found that PHC clinics established after the PHC Choice Reform were located in areas with fewer older adults living alone as well as fewer single parents, but that no significant effects were noted for mean income, percentage of immigrants, education, unemployment and children <5 years. The study concluded that there were some negative effects on geographical equity, but that these were relatively minor [17].

#### Effects on the reimbursement system and its impact on resource allocation – an example from Stockholm County Council

An important aspect of the choice reform is that the location of clinics and the patient’s choice of provider to a large extent determine the allocation of resources in PHC, as resources follow the patient. In addition, the design of the reimbursement system for PHC may further impact on resource allocation. The design of Swedish reimbursement systems in PHC vary between different county councils, but most have a mix of capitation (an annual lump sum per listed individual), and fee-for-service (payment per visit), and a smaller portion of pay-for-performance (connected to meeting certain set targets) [19]. The capitation part may also be adjusted for need of health care, by taking into account the socioeconomic composition or the burden of disease in the population, which is the case in most county councils. In most county councils the capitation part constitutes most (about 80%) of the total reimbursement [19].

In terms of reimbursement systems, Stockholm County Council is an outlier and changed its system of resource allocation markedly with the introduction of the reforms. Since the inception of a purchaser/provider model in health care in the 1990s, the county council for many years operated a need-based resource allocation system in order to distribute resources to match the differing needs of health care in the populations of different geographic areas. The system combined need-adjusted capitation (75%), taking age and socioeconomic composition of the population into account, and fee-for-service (25%), and was in operation until 2007. It resulted in considerable extra resources being allocated to disadvantaged areas, in order to match their greater need of health care [20].

With the implementation of the Choice Reform in Stockholm County Council 2008, this needs-based resource allocation system was abandoned. A new reimbursement system was introduced, with the stated intention of creating equal terms for all providers of PHC (rather than equal terms for patients, as in the previous system). Reimbursement became predominantly based on demand (fee-for-service) (60%), and partly (40%) on the number of listed patients in the population (capitation). The capitation was weighted only by age (higher for persons aged 65 years and above) [21]. The notion of creating equal terms for all providers was criticized, as the variation in burden of disease and socioeconomic composition of listed patients creates very different conditions for PHC providers, and would need to be compensated for [22]. The resulting effect on resource allocation was considerable for PHC clinics in disadvantaged areas, one clinic lost more than 30% of its resources from 1 year to the next. This system was in place from 2008 to 2015, when it was replaced in 2016 by a capitation-dominated (60%) system, partly weighted for socioeconomic composition.

### Effects on the process of PHC – the delivery of PHC

#### Effects on the number of visits

When assessing the outcomes of the PHC Choice Reform, different outcomes should be considered. One frequently used outcome is change in number of visits. It should be noted, however, that average number of visits to primary care may not be a conceptually sound measure of access to care. First, it is not clear whether an increase in visits is a good or a bad outcome in terms of the health of the population. An increase, for instance, could indicate an increase in morbidity in the population, which the increase in visits may or may not match.

Second, more disadvantaged groups are in greater need of health services because of their greater prevalence of ill-health and poorer recovery. They often have higher rates of primary care visits than their more affluent counterparts, but even so, their higher rates of visits may still not match their higher level of need. Assessments of equity of access by socio-economic status, therefore, have to adjust for the higher health care need of more disadvantaged groups before assessing if access is equitable and whether inequalities in access have changed over time. Only a few studies make this latter adjustment. These two provisos need to be borne in mind when interpreting the findings of the following studies.

A first study from Stockholm County Council in 2008 [21] reported both increased number of visits and productivity in general, and a decrease in resources in areas with greater need.). Another study from the Scania (Skåne) Region [23] found that access to PHC increased in all groups, but particularly among high income earners. Studies from Stockholm county council have shown varying results. Overall there has been a 35% increase in the number of visits per adult person from 2005 to 2012 [24]. The number of visits per person has increased in all areas, more among high income earners than among low income earners, but more among those with low education than among those with high education [24]. These studies did not adjust for health care needs. However, another study which made this adjustment found that the rate of increase in the number of visits was significantly lower among persons with greater needs (particularly women) and among men born outside Sweden who live in disadvantaged areas [25].

A recent study of three county councils [26] found an increase from 47 to 55% between 2005 and 2012 in Stockholm county council in the proportion of patients making one or more visits to the doctor. In Region Östergötland the proportion was 47% over the same time; in Region Västra Götaland the proportion was 55%. There was an increase in the number of visits in all three county councils, most pronounced in Stockholm County Council where the average number of visits was higher than in the other county councils. In general, groups with low education or low income had a relatively higher number of visits than other groups [26] (as would be expected from their higher level of healthcare need), but it is not clear in this study if the higher number of visits in more disadvantaged groups matched their higher level of need.

#### Effects on the work of doctors and nurses

Few studies have reported on how the PHC Choice reform and altered reimbursement systems have affected the delivery of PHC, the GP work environment and prioritization of patients. A national survey among 360 public and private PHC managers in Sweden in 2012 [19] found that only 16% of the respondents considered that the current reimbursement system to a great extent promoted the priorities they wanted to work for. The proportion was higher in county councils where capitation reimbursement was weighted by socioeconomic factors and morbidity. Nine out of ten respondents in Stockholm County Council stated that the reimbursement system incentivized short visits. The proportion of respondents who agreed that the current reimbursement system supported a prioritization of patients with great health care needs was lowest in Stockholm County Council. There were considerable differences in responses between public and private PHC managers. For instance, at a national level, 70% of public managers agreed with the statement that the current principles of reimbursement risks crowding out patients with greater health care needs, compared to nearly 54% of private managers. Corresponding figures in Stockholm county council were 89% and nearly 61%, respectively. Nationally, only 20% of all PHC managers agreed that the current principles of reimbursement support a health promoting and preventive way of work [19].

An interview study with 24 managers of publicly owned PHC centres in Gothenburg in 2013 found that the reform was perceived as a rapid change, enforced through financial incentives, and that prioritization conflicts arise between patient groups with different needs, demands and levels of empowerment [27]. While the average number of visits per patient increased, chronically ill patients were considered to be crowded out by healthier and more verbally demanding patients.

An interview study among district nurses in western Sweden [28] in 2013 found that the focus on economic benefit may limit the cooperation and exchange of experiences within and between different care units. This could in turn have a negative impact on the quality of care due to competition between different care providers. The reimbursement system emphasized many short doctor visits and the role of nurses was played down. Underused resources and restrictions on nurses had a negative impact on their job satisfaction and the working environment, and may have affected the quality of care as a result [28].

Another small in-depth interview study among six GPs in Stockholm County Council in 2013 [29]) distinguished between effects of the PHC Choice Reform, which resulted in patients becoming customers rather than patients, and effects of the fee-for-service reimbursement system, which put the focus on performing many short visits among patients with lesser needs, in order to generate income. The prioritization of patients with lesser needs was perceived not to be in line with the intentions of the Health and Medical Service Act [29].

A study of how the PHC Choice Reform had affected health promotion and prevention in PHC in Stockholm County Council found that financial incentives were geared towards producing many visits, at the expense of health promotion and preventive activities, for which there was not time and no reimbursement [30].

Achieving integrated care for persons with greater health care needs is another goal of PHC and requires organized collaboration between PHC and secondary and tertiary health care services, as well as with municipal social services which are responsible for residential care of elderly. A recent government investigation [10] concluded that the PHC Choice Reform has not been conducive, but rather an obstacle, to achieving integrated care. The investigation even suggested that PHC should be divided into two different organizations: one according to the current PHC Choice Reform and another organization for elderly persons with complex health care needs, which should be exempted from the mandatory PHC choice regulations [10].

### Effects on the outcomes of PHC

#### Effects on patient satisfaction

Patient satisfaction has been investigated in several studies after the PHC Choice Reform. One study [31], based on patient survey data from three county councils (Region Scania, Region Halland and Region Västra Götaland), found that the patient perceived quality was lower in larger cities and in clinics with a greater proportion of the listed having more difficult socioeconomic circumstances, but higher among patients with greater need of health care. Private clinics had higher patient ratings than public clinics, but were to a greater extent located in more affluent areas. Patients at clinics with a greater number of listed patients were less satisfied than those with fewer listed patients [31]. Similar results were shown in another report [32].

### Effects on population health

Before the PHC Choice Reform, the responsibility for health of the population in the catchment area lay with the local PHC clinic. With the reform, the assignment of PHC was limited to the listed individuals, which may have an important impact on the role of PHC in improving public health on a population level. Health promotion may be carried out through collaboration with other local agencies such as schools, social services, employment agency or non-governmental organizations, in working with improving health-related behaviors requiring community-wide action, for instance increasing physical activity or reducing smoking. As this is no longer part of the assignment of PHC reimbursed by the county council, there is a risk that such collaboration activities are no longer seen as the responsibility of the local PHC clinic, as found in one study in Stockholm County Council [26].

The government investigation which found that the PHC Choice Reform had increased the difficulties in achieving integrated care among elderly with complex needs [10] suggests that PHC is not organized optimally with respect to elderly patients with complex needs.

## Discussion

### Effects on the structure of PHC

In order to follow up the impact of the PHC Choice Reform, different indicators may be studied. In terms of the impact of the PHC Choice Reform on the structure of PHC, it is evident that the reform has increased the number of PHC clinics and the average number of visits to PHC, but it is debated whether that is a good indicator of improvement in access to health care in its wider sense. The evidence reviewed in this paper indicates that increases in number of visits have not been uniform across the population. The National Audit Office concluded that more new clinics had established in already well served areas, and in interviews PHC providers indicated they were unwilling to establish practices in areas with high levels of need for care, even if reimbursement systems were to take patients’ need of care into account (17). However, the study by Isaksson et al concluded that there were some, but only minor, negative effects on equity (16). It is difficult to measure the supply and access to care in correct ways, and on a meaningful area level.

The National Audit Office report suggests that PHC providers have been choosing their patients, rather than patients choosing their PHC provider, which is actually the reverse of what the PHC Choice Reform was designed to do. If this conclusion holds, it would be an example of Julian Tudor Hart’s classical ‘inverse care law’ about the operation of market forces in health care, as stated in his 1971 seminal Lancet paper [33]: “The availability of health care tends to vary inversely with the need for it in the population. This inverse balance operates more completely where health care is most exposed to market forces, and less so with less exposure.”

A crucial impact of the PHC Choice Reform, as evidenced particularly in Stockholm County Council, is a change in resource allocation to the detriment of areas with greater health care needs, through the combined effect of the PHC Choice Reform itself, the fee-for-service dominated reimbursement system without socioeconomically weighted capitation which was in place 2008 − 2015, and a difference in health-seeking behavior and demand for health care between different areas.

**Fig. 1:**
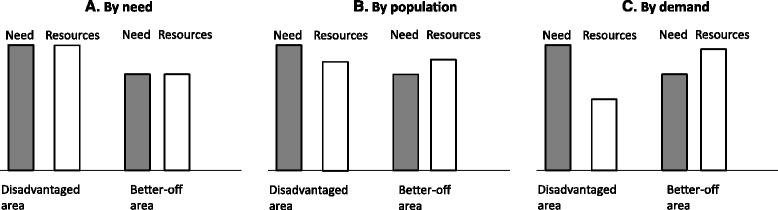
Effects of resource allocation by different principles (**a** need, **b** population, **c** demand) on two populations of the same size with different levels of need (adapted from Hung et al. [37]

An illustration is given in Fig. 1, comparing two areas with the same population size, but with different composition of the population with respect to need. If resources are allocated according to need (A), the population in disadvantaged areas will receive more resources than the population in better-off areas. This was the case in the former need-based reimbursement system in Stockholm County Council in place until 2008, in line with the intentions of the Swedish Health and Medical Services Act [1].

However, if resources are distributed only by population size (B), the disadvantaged area will receive less resources in relation to the level of need in that area, and the population in the better-off area will receive more resources in relation to the area’s level of need. This situation corresponds to the capitation part of the fee-for-service dominated reimbursement system with no socioeconomic weighting, disregarding population differences in need.

If resources are allocated by demand for health care services (C), the population in the better-off area is likely to get more resources than the population in the disadvantaged area, because their demand for services is greater, in spite of their relatively lesser need of health care services [34]. This situation may represent the effect of the fee-for-service component in the reimbursement system in Stockholm County Council, which generates an increase in the number of visits, as fee-for-service incentivizes many short visits, generated both by demand of patients and by doctors prioritizing such visits.

In this manner, an adverse outcome of the PHC Choice Reform and a demand-oriented reimbursement system, may be a reallocation of resources away from areas with greater need to areas with less health care needs, as evidenced in Stockholm County Council [29].

### Effects on process – delivery of PHC

The evidence suggests that the PHC Choice Reform has increased the number of visits to PHC. However, some reports indicate a greater increase among groups with lesser health care needs than among those with greater health care needs. According to some studies the reform appears to have had a negative impact on the process and delivery of PHC, as evidenced by the studies of PHC managers and nurses [19, 23–25]. These studies provide an account of how employees in publicly owned PHC clinics perceive the PHC Choice Reform and changes in reimbursement systems, with a shift in prioritization of patients towards those with lesser needs, and an increased focus on the role of the doctor. As indicated in the national survey of PHC managers, those working in privately owned PHC clinics were more positive to the reform and changes in reimbursement systems than those working in publicly owned clinics [19]. This is an important aspect to study in further detail.

### Effects on outcomes of PHC

One key finding concerns the impact on integrated care: that the PHC Choice Reform is considered by providers to be an obstacle in organizing integrated care, particularly for elderly with complex needs of care [10]. This is important not least because elderly with complex needs of care are a large and important group of patients in PHC, and the finding is in stark contrast to the intentions of the Health and Medical Services Act [1]. The PHC Choice Reform and change in reimbursement system was also considered by doctors and nurses in Stockholm county council to have reduced the emphasis on health promotion and prevention, because of the focus to produce many visits and generate income to the clinic [26]. The responsibility of PHC for population oriented health promotion activities has also been reduced, because of the focus only on listed patients after the reform, which may further reduce the population health impact of PHC [26].

The interaction between the PHC Choice Reform and simultaneous changes in the reimbursement system provides further difficulties in interpreting the findings of evaluations. The greater increase in the number of visits observed in Stockholm County Council, for example, may be related to the fee-for-service based reimbursement system. In most of the reviewed studies, the number of patient visits to doctors was the measured outcome. However, as this measure depends on the reimbursement system, it may have severe shortcomings. As indicated in some of the referenced studies, the available statistics on the number of visits do not reflect the content or quality or potential effect on health of the visit. As the goal of health care is to improve health, there is a need to go beyond measures of productivity, such as number of visits, to actually measuring the impact on health status improvement, by using patient-reported outcomes [35]. In Stockholm county council there is anecdotal evidence that previously longer visits were divided into several shorter, in order to gain revenue, because of the reimbursement system which gave the same amount for a short as for a longer visit. In some of the studies the increase in number of visits has been greater among those with lesser needs of care [28, 30], in line with an earlier review study of choice reforms in health care in European countries [36].

## Conclusion

In conclusion, the evaluative evidence is sparse and incomplete. The studies to date indicate that the PHC Choice Reform, as implemented by some county councils, predominantly Stockholm, increased access to PHC and increased the average number of visits to PHC, but seems to have particularly benefitted those in more affluent groups and with lower health care needs. In addition, it has made integrated care for those with complex needs more difficult. Among GPs and nurses in PHC there are mixed opinions about the reform. Some consider that persons with greater needs are not prioritized; others are more positive. Resource allocation to PHC has become more dependent on provider location, patient choice and demand, and less on need of care. In view of the more restricted assignment there is also a risk of a reduced impact on population health of PHC. On the available evidence, the PHC Choice Reform may have damaged equity of primary health care provision, contrary to the tenets of the Swedish Health and Medical Service Act. This situation needs to be carefully monitored and countered where necessary. Further studies are needed to follow up the long-term impacts of the reform on the structure, process and outcomes of PHC in Sweden and how different types of reimbursement systems may moderate these impacts.

## Abbreviations

GP: General Practitioner
PHC: Primary Health Care
